# Sex, Knowledge, and Attitude of Stroke Survivors Attending Bebe Herbal Center on Risk Factors before and after Stroke

**DOI:** 10.1155/2021/6695522

**Published:** 2021-04-28

**Authors:** Polycarp U. Nwoha, Florence O. Okoro, Emmanuel C. Nwoha, Fidelia N. Chukwu, Chidinma O. Nwoha, Nkeiru C. Ogoko, Peace N. Nwoha, Chika A. Idaguko, Augustine U. Obi, Ezenna M. Agwu, Iyanu O. Ayoola, Sunday O. Osonwa, Ifeoma H. Okpara

**Affiliations:** ^1^Department of Anatomy and Cell Biology, Obafemi Awolowo University, Ile-Ife, Nigeria; ^2^Centre for Scientific Investigations and Training, Owerri, Imo State, Nigeria; ^3^School of Nursing, College of Health and Human Services, University of North Carolina at Charlotte, USA; ^4^Bucks New University, Alexandra Road, High Wycombe, UK; ^5^Health Centre, University of Medical Sciences, Ondo City, Ondo State, Nigeria; ^6^Military Hospital, 82 Division, Enugu, Enugu State, Nigeria; ^7^Edo State University, Iyamho, Edo State, Nigeria; ^8^Department of Anatomy and Neurobiology, Imo State University, Owerri, Nigeria; ^9^Mother Theresa Hospital, Aba, Abia State, Nigeria; ^10^Liviashammah Hospitals Ltd., 5 Shammah Close, Abuja-Keffi Road, Maraba, Nasarawa State, Nigeria

## Abstract

**Objective:**

The objective of this study was to investigate the extent stroke survivors who attended an herbal center knew of stroke risk factors and whether significant sex differences existed. *Study Design*. This was a cross-sectional study conducted from January to June 2018 at Bebe Herbal Center, and it involved two well-trained assistants who interviewed 149 first-time stroke survivors after consent and ethical approval were obtained. The survivors self-reported their knowledge, attitude, and beliefs on risk factors before and after stroke. *Statistical Analyses*. Means of continuous variables were compared using Student's unpaired *t*-test, while categorical variables between males and the females were analyzed using Pearson's chi-square test. *P* < 0.05 was taken as significant.

**Results:**

Mean age of men (64.81 ± 1.24 yrs) was significantly higher than that of women (61.39 ± 1.42 yrs) (*F* = 0.096, *t* = 1.79, df = 147; *P* < 0.05). More men than women were 60 years and above while more women than men were below 60 years. Pearson's chi-square test showed significant association of sex with education (*χ*^2^ = 12.31; df = 3, *P* < 0.006), occupation (*χ*^2^ = 23.65; df = 4, *P* < 0.001), alcohol intake (*χ*^2^ = 24.23; df = 1; *P* < 0.001), and smoking (*χ*^2^ = 9.823; df = 1; *P* < 0.001). The commonest risk factor suffered was hypertension (73.1%), followed by alcohol intake (59.1%), smoking (31.5%), and diabetes mellitus (26.7%); these affected men more than women. Male survivors unaware of their hypertensive status were more likely to have stroke than females, and age had a significant effect on the likelihood of developing a stroke; the same was occupation.

**Conclusions:**

These survivors suffered mainly from hypertension, triggered by psychosocial problems and diabetes mellitus; their stroke seemed fueled by unrecognized hypertension, unrecognized diabetes mellitus, ignorance of hyperlipidemia, and wide-scale belief in witchcraft as risk factor. Awareness programs in the third world should take these observations into consideration.

## 1. Introduction

Stroke is a noncommunicable disease that is fast devastating the human population. It is regarded as the leading cause of death and disability in the world [1] and the second leading cause of death in developed countries, after ischemic heart disease [2]. The burden of stroke has shifted from the developed to developing countries [3] and has become the leading cause of death in sub-Saharan Africa and Latin America [4–6]. It accounts for 7%-9% of all deaths in Africa [7]. While its occurrence is decreasing in the developed countries, it is increasing at an alarming rate in the developing ones [8, 9]. Currently, 75% of all stroke deaths occur in developing countries [3] while the WHO predicted that by 2030, 80% of all stroke cases will be in low- and middle-income countries [10]. Still, these developing countries are overburdened by very harsh economic conditions and ravaging endemic diseases. And one of the important things needed to change the trend is modification of lifestyle by ensuring that people abandon behaviors that predispose to stroke. Awareness programs come in handy for the high-risk group, especially those who have suffered a first stroke, because a second stroke which they are prone to could be more devastating [11, 12].

Numerous studies on community knowledge of stroke risk factors have been carried out in western countries [13] and in sub-Saharan Africa [9, 14]. Such studies have also been extended to stroke survivors attending hospitals in developed and developing countries [15]. However, there is a dearth of information on awareness programs to survivors attending herbal centers. This is despite the fact that most stroke patients in developing countries, including Nigeria [16] and South and East Asia [17], for one reason or the other, patronize herbal centers [18]. The interest in traditional healers and herbal medicine is so high that most stroke patients would have visited them first before going to a hospital or the reverse [14]. This lack of awareness programs may be contributing to the increasing rate of stroke cases in developing countries, especially in sub-Saharan Africa. For survivors to benefit maximally from any awareness program, it is important to first ascertain their mindset towards stroke in order to correct any wrong beliefs and harmful attitudes. It is equally important to know how these factors are influenced by sex. The present work, therefore, investigated the knowledge, attitude, and beliefs of male and female stroke survivors attending an herbal center towards stroke risk factors.

## 2. Methods

### 2.1. Study Population and Ethical Approval

This study was conducted at Bebe Herbal Center located in Umunomo Ihitteafoukwu, a rural community in Ahiazu Mbaise Local Government Area of Imo State, Nigeria, from January to June 2018. Ahiazu Mbaise is an agrarian community with a population of 227,400 people and surrounded by other densely populated local government areas. Bebe Herbal Center is a traditional herbal home that predominantly manages stroke cases from all walks of life. It has outpatient facilities and attends to patients three times a week—Mondays, Wednesdays, and Saturdays. It manages some other ailments including kidney problems, liver problems, and diabetes mellitus. The participants were first-ever stroke survivors, assisted by their caregivers, who could recall their lifestyles before and after the stroke. Those with insufficient information were dropped from the study. Ethical approval was obtained from the Ethical Committee of the Center for Scientific Investigation and Training, Owerri, Nigeria. Participants gave oral and written informed consent before data collection.

### 2.2. Investigation

We conducted a cross-sectional study involving those who were first-ever stroke survivors, attended Bebe Herbal Center for a minimum of two weeks, and provided an adequate response to questions or had caregivers to help with such intimate answers. We excluded those who were attending for the first time because of a lack of enough information. Other clinical pictures presented by the patients were speech defects and facial anomalies.

Two trained assistants interviewed the patients using a validated structured questionnaire with open-ended questions. The open-ended questions allowed the patients to state exhaustively and without bias what they knew about themselves and their stroke condition. The trained research assistants deduced the necessary information from their narrative. Open-ended questions were as follows:

1. Tell us about yourself
2. Tell us your health history before the stroke
3. How did your stroke happen?
4. What were the things that could have contributed to your stroke?
5. From your experience, what advice would you give nonsufferers to prevent a stroke?

Where necessary, clarifications were given by asking specific questions such as the following: Were you hypertensive before the stroke? When did you know you were hypertensive—before or after the stroke? If before the stroke, were you on medication, and was it regular? When did you know you had diabetes? If before the stroke, were you on medication? They were also asked if they had any heart disease, kidney problems, liver problems, smoked cigarettes, drank alcohol, engaged in regular physical activity, had a family history of stroke, and diagnosed with high cholesterol? Generally, the respondents were allowed to mention multiple precipitating factors they were aware of.

Information gathered from the interview enabled us to compute the sex, age, and other demographic features.

### 2.3. Data Analysis

Data obtained from the responses were arranged into variables and presented as frequencies and percentages for categorical variables. Means of continuous variables were compared using Student's unpaired *t*-test whereas categorical variables between males and females were analyzed using Pearson's chi-square test. *P* values less than 0.05 were considered significant. The statistical package used was SPSS version 23.0.

## 3. Results

A total of 161 survivors consented to the study and were interviewed. Twelve cases were excluded due to incomplete information. In the final analysis, 149 cases were included, giving a response rate of 92.5%.  Table 1 shows the demographic and clinical characteristics of the sample, according to sex. Men were 88 (59.1%) and women 61 (40.9%), with a sex ratio of 1.44 : 1. Mean age for the participants was 63.41 ± 11.53 years (range: 28 to 87 years); the difference was significant (*P* = 0.05). The highest number of survivors was within the 70- to 74-year age group; men outnumbered women after age 60, whereas the contrary was observed under age 60. Pearson's chi-square test showed that education had a strong significant association with sex (*χ*^2^ = 12.31; df = 3, *P* < 0.006). More men than women had primary education, secondary education, and tertiary education. Few (4.7%) did not have formal education, giving a literacy level of 95.3%. Occupation had a strong significant association with sex (*χ*^2^ = 23.65; df = 4; *P* < 0.001) with more men than women being unemployed, artisans, and civil servants while women more than men were traders and farmers. The remaining variables, side of body affected, number of children in a family, and age group, did not have significant association with the sex of the survivors (*P* > 0.05).

Table 2 shows the frequency of risk factors in our sample. The commonest risk factor encountered by the survivors was systemic hypertension, followed by alcohol intake, tobacco smoking, and diabetes mellitus; in all these, men outnumbered women. Only half of survivors were aware of their hypertensive status before stroke and one quarter their diabetic status. Fifteen survivors were unaware of their hypertensive status, even after their stroke event, while 28 (18.4%) were unaware of their diabetic status, even after stroke. There was no association between awareness of hypertension or diabetes with sex. Pearson's chi-square test showed a significant association of alcohol intake (*χ*^2^ = 24.23; df = 1; *P* < 0.001) and cigarette smoking (*χ*^2^ = 9.823; df = 1; *P* < 0.001) with sex. More men than women ever drank alcohol (24.8% vs. 14.1%) and ever smoked a cigarette (24.8% vs. 6.7%). None engaged in heavy alcohol consumption or chain smoking. Three respondents had their stroke after they were confronted with very sad incidents.

Table 3 shows the response of survivors to what they thought precipitated their stroke and their pieces of advice to nonsufferers to prevent a stroke. There was no significant association of the variables with sex (*P* > 0.05). Twenty-nine (19.4%) survivors said their stroke was caused by hypertension. Diabetes mellitus, alcohol intake, family history, poor diet, and overweight were mentioned separately by a total of seven (4.7%) persons. Twenty persons said their stroke was caused by psychosocial stress (worry) and another twenty spiritual attacks (witchcraft/diabolical influence), while a high number had no knowledge of what precipitated their stroke. To the question of what advice they could give others who do not have a stroke, ten advised checking of blood pressure, 12 medical check-up, six advised against alcohol, sugar intake, and 15 persons advised prayers to God. A large number said they had no advice to give. The other established important risk factors such as smoking, hyperlipidemia, atrial fibrillation, HIV infection, TIA, and physical inactivity were not mentioned by any survivor.

## 4. Discussion

Generally, the findings have shown that most survivors who attended Bebe Herbal Center had little or no knowledge of stroke risk factors and were not bothered. The reason for this ignorance and apathy cannot be attributed to the location of Bebe Herbal Center or to the assumption that most stroke cases in developing countries are rural dwellers, poor, and uneducated [19, 20]. Bebe Herbal Center attracts stroke patients from urban and rural areas, rich and poor in Nigeria [9, 16], and a high literacy level of 95.3% was recorded among participants in this work. Social status and education level, therefore, could not be contributing factors. Poor knowledge of risk factors among stoke survivors has also been reported in works in developed countries [21, 22]. The reason for this may be traced to depression, because stroke can lead to depression [23]. The general lack of interest was even more glaring in the present work where most of the survivors remained unaware of their hypertensive and diabetic status even after their stroke event. Of the ten modifiable risk factors (hypertension, lack of physical activity, abnormal lipids, unhealthy diet, abdominal obesity, psychological factors, current smoking, cardiac causes, heavy alcohol consumption, and diabetes mellitus) known to be associated with stroke in developed and developing countries plus infectious diseases peculiar to Africa [24], the survivors could mention only four (high blood pressure, worries, excessive alcohol consumption, and sugar/diabetes mellitus). It is important that stroke survivors become interested in their health because people with first-ever stroke run the risk of a second stroke within five years, resulting in high mortality and increased disability [23]. Poor knowledge has been reported in previous works conducted in communities and hospitals in developed and developing countries [25, 26]. Lack of knowledge of stroke risk factors contributes to the rising incidence of stroke among Africans [27, 28]. This drawback should be addressed with intensive health education campaigns, emphasizing the need for regular checking of blood pressure, blood sugar, and blood lipids and made attractive by making access to monitoring devices easily available. Stroke poses a serious economic burden beyond the reach of most victims [29]. Its awareness, therefore, should be intense and extended to the herbal centers as they have become the first choice and maybe the preferred choice for most stroke cases in Nigeria and other developing countries [9].

The mean age of 63.4 years reported here is similar to what was reported by previous authors in Nigeria [30–32] but higher than the values reported in South Africa [20]. Overall, the mean age of stroke in developing countries is lower than in developed countries [24, 33, 34]. Part of the reason for this is lower life expectancy and inadequate emergency health facilities for acute stroke care in developing countries, resulting in higher case fatalities [19]. The highest concentration of stroke cases in this study was in the 70- to 74-year age group, similar to findings by other authors in Nigeria [5, 35] and outside Nigeria [23]. In Nigeria, the middle age group is a time of intense responsibility and attendant psychosocial stress and pressure. Nine of the survivors in the present work were below 40 years of age, a number lower than 11.9% and 10-12% reported elsewhere in Nigeria [30, 35], but higher than 3.5% reported in Egypt [36]. Some researchers have, however, stated that 10-20% of all stroke cases in the tropics occur in the young age below 40 years [35]. The lower value recorded in the present work could mean that fewer young men with stroke visit the herbal center. It could also mean that they would have recovered from their condition before thinking of alternative therapy, supporting the report that those below 50 years of age recover from stroke fast [23]. Our study is the first of its kind in an herbal center. The percentage of strokes reported for young Africans is very much lower than the 30% reported in Karachi, Pakistan [37], suggesting that stroke is not much of a health issue among young Africans.

We reported that more women were below and more men were above 60 years. This is the first report of this essential finding regarding stroke variation with age and sex and will be helpful in planning stroke awareness among different age groups. A plausible explanation for this variation is the different psychosocial responsibilities confronting women and men in Nigeria. Most of the women in this work were below 60 years and widowed, as such had the undue responsibility of taking care of their families alone, a hostile situation that could heighten psychosocial problems, leading to high blood pressure and maybe stroke. After 60 years, most of the men retired without pensions and gratuities to support families, also leading to increased psychosocial stress, high blood pressure, and stroke. It has also been reasoned elsewhere that the lower number of women in the higher category could be due to the higher death of women with increasing age [38]. The lower number recorded for those below 40 years and the high number for those above 70 years in this work suggest that many of the older people visit herbal centers. This realisation has to be taken into account in planning the awareness programs for stroke patients attending herbal centers.

We showed that systemic hypertension was the commonest risk factor suffered by 83.2% of the 149 survivors, followed by diabetes mellitus (45.6%). It is also the most prevalent risk factor in other works conducted in Nigeria and sub-Saharan Africa [34, 39], Iran [40], and the INTERSTROKE study of 2016 involving 32 countries [41]. It is clear that in Nigeria, hypertension, diabetes mellitus, and hyperlipidemia are the key risk factors that predispose to stroke. Attention should therefore be focused on tackling the direct and indirect causes of hypertension. This advice becomes essential when it is realised that most stroke survivors in this work had unrecognized hypertension and unrecognized diabetes mellitus, with many still unaware of their hypertensive and diabetic status, even after their stroke event. It has been noted elsewhere that hypertension is dominant and present in 79% of all cases of stroke with a great majority of victims not knowing their blood pressure status [14, 30]. This poor attitude is irrespective of educational level. Unrecognized hypertension (18.7%) and unrecognized diabetes mellitus (4.2%) have also been reported among stroke survivors in the Stroke Belt of the United States [15]. A consequence of this neglect is recurrent stroke, which may lead to death (25% within 28 days) or increased risk of further disability, dependence, and institutionalisation [23]. Serious attention should be paid to hypertension in Nigeria as cases of stroke are rising exponentially [42, 43], demanding a declaration of stroke as an emergency. The report that three respondents had a stroke after being confronted with very sad news suggests that they might have experienced a very sudden spike in blood pressure at that moment, which could have compromised the integrity of the blood vessels, leading to maybe hemorrhagic stroke. The drawback to discerning stroke type in this work is the lack of CT or MRI facilities in the hospitals visited by the survivors. Nonetheless, people should check the manner they react to sudden sad news to avoid precipitating a stroke.

It was also reported here that more than half of the survivors never smoked cigarettes or inhaled snuff, and this number is nearly thrice those who used tobacco (cigarettes or snuff). Even many of the small number who smoked had quit before their stroke incident happened and there was no chain smoker among them. Smoking was, therefore, not an issue among this group; yet it is better not to smoke because passive or active smoking is known to increase the risk of acute stroke [11, 38]. It is also beneficial to quit smoking if one is a current smoker as one work did not show a statistically significant difference between ex-smokers and those who never smoked; ex-smokers who had quit for 10 years had no increased risk of stroke [38].

In the present work, slightly more than half had consumed alcohol at one time or the other before stroke. Many of the drinkers had quit alcohol even before their stroke incident, thus suggesting that alcohol consumption was not an issue among the survivors; and none of the previous or current drinkers was a heavy drinker, a known risk factor for stroke [4, 11]. An awareness study of stroke risk factors among stroke-free people living in Accra, Ghana, revealed that the commonest risk factor cited by the respondents was alcohol intake while the least was diabetes mellitus [9], showing very poor knowledge of risk factors among this group.

When asked about the things that could precipitate a stroke, none of the survivors mentioned hyperlipidemia. Yet a study in Kano, northern Nigeria, showed more than half of the stroke participants suffered from hyperlipidemia [44]. In America, hyperlipidemia is the leading risk factor for stroke, where 1 in every 3 adults is affected [45]. No mention was also made of atrial fibrillation (cardiac disease), which is also a common risk factor for stroke in this environment [4]. Rather, several mentioned psychosocial stress (worries) and spiritual affliction (diabolical attack) as the main predisposing factors for stroke. These responses are very typical here in Nigeria, Ghana, Uganda, and other countries of sub-Saharan Africa leading to high patronage of traditional healers and herbal centers for the management of stroke [9, 46]. Psychosocial problems seem to be self-inflicted among Nigerians as most of them have large families to worry about in the midst of very harsh economic conditions, ravaging poverty and endemic diseases. One hundred and seven survivors in the present work had 5 to 8 children per family. This puts a lot of pressure as families struggle to train their children, most times without government or external support. Thus, on a daily basis, they are subjected to survival stress that could accumulate to high blood pressure and subsequent stroke. Families should have the adequate number of children they could comfortably cater for. Psychological stress, therefore, is the number one indirect risk factor for stroke in Nigeria and sub-Saharan Africa. It should be seriously addressed in every stroke awareness program. Another serious peculiar factor for people in this part of the world is the belief that whatever disease condition that is unexplainable is caused by witchcraft (spiritual or diabolical means) and this includes stroke. Instead of seeking proper medical advice and treatment, they resort to native doctors or prayer houses, thus delaying treatment and complicating health issues.

Regarding proper advice to nonsufferers to prevent stroke, a low number mentioned only one of the following four risk factors—avoid high blood pressure, excessive alcohol consumption, excessive sugar consumption, and overweight—while an appreciable number had no advice to give, implying that they learnt nothing from their condition that could be usefully shared with nonsufferers to prevent a stroke. It is a general belief that experience is a good teacher and that others are bound to believe you and learn from you if the advice comes from a survivor. On the other hand, their ignorance could lead to false knowledge and teachings about predisposing factors, as depicted in their responses, where their pieces of advice included staying away from diabolical people, praying, and visiting Bebe Herbal Center. The ultimate effect of all these misleading beliefs is that stroke cases will continue to rise in Nigeria and sub-Saharan Africa. For any stroke prevention campaign in Nigeria, sub-Saharan Africa, and the third world to be effective, there is a need to firstly address the wrong beliefs, especially the strongly held belief that diabolical causes are responsible for most stroke cases.

Pearson's chi-square test showed that sex is associated with the occupation of stroke survivors; male civil servants have more likelihood of having a stroke than females, while female farmers or traders have more likelihood of having a stroke than their male counterparts. In Nigeria, civil service has a preponderance of males than females while trading and farming occupations have a preponderance of females than males [47]. The two occupations, civil service and trading, have also the tendency to sedentary life as there is no provision for recreational activities, leading to an increased tendency for stroke [48]. Furthermore, a situation where civil servants are owed salaries, gratuity, or pensions, as often happens in Nigeria, puts them into serious psychosocial trauma which most often triggers high blood pressure. Traders, equally, are often subjected to hostile environments for their businesses, keeping them anxious most of the time, and this could lead to high blood pressure [49]. Farmers in Nigeria are mostly peasants; working for subsistence living always subjects them to serious physical stress, coupled with the anxiety of not having enough to take care of their large families. People in these occupations should be properly advised against psychosocial stress to minimise the incidence of high blood pressure and resultant stroke. Awareness programs should be intensified as studies have shown that increased awareness of stroke risk factors among people at high risk for stroke leads to increased compliance with stroke prevention practices [50].

## 5. Strengths and Limitations

This is the first reported study to be undertaken among stroke participants in an herbal center. Considering the fact that stroke occurrence and deaths are increasing alarmingly in the third world and that most stroke patients patronize herbal centers, the findings in this study will be essential in enhancing stroke education and influencing policymakers to restrategise in combating the devastating effects of stroke. A limitation of this work is that it is a cross-sectional one that took place in one herbal center and so the results obtained cannot be easily generalized to the larger community. However, it affords great insight for planning for high-risk stroke patients in general. It calls for holistic planning strategies targeting herbal centers as well as hospitals. Another limitation is the inability to differentiate subtypes of stroke due to lack of proper imaging facilities. A third limitation is the self-reported response and lack of confirmation of the claims with neurodiagnostic tests and medical records. However, no one who does not have a stroke would claim he has. Such self-reporting has also been known to be sensitive, specific, and reliable [51, 52].

## 6. Future Directions and Conclusions

Stroke affected mostly the middle-aged group, more women below 60 years than men, and the older age patronized herbal centers more than the young age below 40 years. Hypertension was the commonest risk factor and seemed triggered by psychosocial problems that are rampant in Nigeria. Educational programs should dispel the harmful, erroneous, and widely held belief that witchcraft causes stroke, and people should be made to regularly check their blood pressure, blood sugar, and blood lipids and engage in physical activities. In developed countries, stroke patients patronize hospitals but not so in sub-Saharan Africa. It becomes important to understand why stroke patients in sub-Saharan Africa are skeptical about hospitals and so be able to utilise the advantages of both hospitals and herbal centers to effectively combat stroke in developing countries.

## Figures and Tables

**Table 1:** Descriptive characteristics of the survivors and Person's chi-square test between male and female.

Variables	Male	Female	*χ* ^2^
	*n* (%)	*n* (%)	
*Age group (yrs)*			**12.04**
<45	6 (4.0)	3 (2.0)	
45-49	3 (2.0)	2 (1.3)	
50-54	6 (4.0)	12 (8.1)	
55-59	9 (6.0)	9 (6.0)	
60-64	14 (9.4)	8 (5.4)	
65-69	7 (4.7)	9 (6.0)	
70-74	27 (18.1)	9 (6.0)	
75-79	10 (6.7)	7 (4.7)	
≥80	6 (4.0)	2 (1.3)	
*Education*			**12.31** ^∗^
Primary school	41 (27.5)	38 (25.5)	
Secondary school	25 (16.8)	14 (9.4)	
Tertiary school	19 (12.8)	5 (3.4)	
No school	3 (2.0)	4 (2.7)	
*Occupation*			**23.65** ^∗^
Unemployed	4 (2.7)	1 (0.7)	
Trader	18 (12.1)	23 (15.4)	
Artisan	29 (19.5)	11 (7.4)	
Farmer	11 (7.4)	21 (14.1)	
Civil servant	26 (17.4)	5 (3.4)	
*Side affected*			**0.403**
Left	52 (34.9)	32 (21.5)	
Right	36 (24.2)	29 (19.5)	
*Number of children*			**1.146**
No child	4 (2.7)	3 (2.0)	
1-4	19 (12.8)	16 (10.7)	
5-8	56 (37.6)	35 (23.5)	
Above 8	9 (6.0)	7 (4.7)	

^∗^ *P* < 0.001.

**Table 2:** Stroke risk factors encountered by the survivors and Pearson's chi-square test of association with sex.

Variables	Male (*n*, %)	Female (*n*, %)	*χ* ^2^
*Hypertension*			3.68
Aware before stroke	44 (29.5)	36 (24.2)	
Aware after stroke	16 (10.7)	13 (8.7)	
No knowledge	12 (8.1)	3 (2.0)	
Not hypertensive	16 (10.7)	9 (6.0)	
*Diabetes mellitus*			**0.082**
Aware before stroke	22 (14.8)	16 (10.7)	
Aware after stroke	02 (01.3)	0	
No knowledge	17 (11.4)	11 (7.4)	
Not diabetic	47 (31.5)	11 (7.4)	
*Alcohol intake*			**24.23** ^∗^
Yes	67 (45.0)	21 (14.1)	
Never	21 (14.1)	40 (26.8)	
*Cigarette smoking*			**9.823**
Yes	37 (24.8)	10 (6.7)	
Never	51 (34.2)	51 (34.2)	

^∗^ *P* < 0.001.

**Table 3:** Survivors' knowledge of causes of stroke, their advice for prevention, and Pearson's correlation with sex.

Variables	Male	Female	*χ* ^2^
	*N* (%)	*N* (%)	
*Risk factors of stroke*			12.04
Hypertension	20 (13.4)	9 (6.0)	
Diabetes mellitus	0 (0.0)	1 (0.7)	
Alcohol	3 (2.0)	0 (0.0)	
Family history of stroke	1 (0.7)	0 (0.0)	
Psychosocial stress	8 (5.4)	12 (8.1)	
Spiritual attack	13 (8.7)	7 (4.7)	
Normal sickness	9 (6.0)	5 (3.4)	
Poor diet	0 (0.0)	1 (0.7)	
Overweight	0 (0.0)	1 (0.7)	
Do not know	34 (22.8)	25 (16.8)	
*Advice to prevent stroke*			**11.782**
Check blood pressure	4 (2.7)	6 (4.0)	
Pray to God	8 (5.4)	7 (4.7)	
Avoid stress/worry	11 (7.4)	10 (6.7)	
Avoid alcohol	2 (1.3)	1 (0.7)	
Medical check-up	7 (4.7)	4 (2.7)	
Good behavior	9 (6.0)	3 (2.0)	
Avoid sugar	1 (0.7)	2 (1.3)	
Visit Bebe center	2 (1.3)	1 (0.7)	
Avoid diabolical people	1 (0.7)	2 (1.3)	
Avoid overweight	2 (1.3)	1 (0.7)	
No advice to give	35 (23.5)	30 (20.1)	

## Data Availability

Data are available with the corresponding author on request.
